# The Effectiveness of Oxytocin for Preventing Postpartum Haemorrhage: An Individual Participant Data Meta‐Analysis

**DOI:** 10.1111/1471-0528.18279

**Published:** 2025-07-16

**Authors:** Madeline Flanagan, Arsheeya Rattan, Ling Shan Au, Malitha Patabendige, Carole‐Anne Whigham, Suze Jans, Patience Cofie, G. Justus Hofmeyr, Leiv Arne Rosseland, Andrew Weeks, Wentao Li, Ben W. J. Mol

**Affiliations:** ^1^ Department of Obstetrics and Gynaecology Monash University Melbourne Victoria Australia; ^2^ Department of Obstetrics and Gynaecology Monash Health Melbourne Victoria Australia; ^3^ Department of Child Health TNO Leiden the Netherlands; ^4^ PATH Accra Ghana; ^5^ Effective Care Research Unit, University of the Witwatersrand and Walter Sisulu University East London South Africa; ^6^ Department of Obstetrics and Gynaecology University of Botswana Gaborone Botswana; ^7^ Faculty of Medicine, Institute of Clinical Medicine University of Oslo Oslo Norway; ^8^ Department of Research and Development, Division of Emergencies and Critical Care Oslo University Hospital Oslo Norway; ^9^ Department of Women's and Children's Health The University of Liverpool Liverpool UK; ^10^ National Perinatal Epidemiology and Statistics Unit, Centre for big Data Research in Health and School of Women's and Children's Health The University of New South Wales Sydney New South Wales Australia; ^11^ Aberdeen Centre for Women's Health Research Institute of Applied Health Sciences, School of Medicine, Medical Sciences and Nutrition, University of Aberdeen Aberdeen UK

**Keywords:** meta‐analysis, oxytocin, postpartum haemorrhage, randomised controlled trial

## Abstract

**Background:**

Post‐partum haemorrhage (PPH) is a common complication of labour.

**Objective:**

To assess the effectiveness of oxytocin in comparison to no treatment for preventing PPH.

**Selection Criteria:**

Published and unpublished randomised controlled trials (RCTs) comparing systemic oxytocin to placebo or no intervention for preventing PPH were included. We did not apply language restrictions.

**Search Strategy:**

We identified RCTs from the Cochrane network meta‐analysis on uterotonics for preventing PPH and updated the search via: Ovid MEDLINE, Embase via Ovid, Web of Science, CENTRAL, CINAHL Plus and clinicaltrials.gov.

**Data Collection and Analysis:**

An Individual participant data (IPD) meta‐analysis.

**Main Results:**

Of 14 eligible RCTs, four provided IPD (*n* = 4304; 51.7% received oxytocin and 48.4% received placebo or no intervention). Meta‐analysis of IPD showed that oxytocin decreased the risk of PPH ≥ 500 mL (aOR 0.59; 95% CI 0.46 to 0.74) and PPH ≥ 1000 mL (aOR 0.51; 95% CI 0.32 to 0.80). Of 10 RCTs that did not share data, seven met trustworthiness criteria while three did not. Trustworthy IPD and aggregate data (AD) from RCTs meeting trustworthiness criteria (*n* = 6003) showed that oxytocin significantly reduced the rate of PPH ≥ 500 mL (aOR 0.53; 95% CI 0.45 to 0.62) and PPH ≥ 1000 mL (aOR 0.59; 95% CI 0.48 to 0.71). RCTs not meeting trustworthiness criteria reported a larger risk reduction of oxytocin for PPH ≥ 500 mL (*n* = 1027; aOR 0.37; 95% CI 0.03 to 4.03) and PPH ≥ 1000 mL (*n* = 1157; aOR 0.13; 95% CI 0.01 to 1.45).

**Conclusions:**

Prophylactic oxytocin reduces the risk of PPH ≥ 500 mL and PPH ≥ 1000 mL compared to no treatment. Twenty‐one percent of RCTs did not meet our pre‐defined trustworthiness criteria, underlining the importance of integrity assessment in evidence synthesis.

## 1 Introduction

Postpartum haemorrhage (PPH) is the leading cause of maternal morbidity and mortality worldwide [1]. Annually, 14 million women experience PPH, resulting in 70 000 maternal deaths. The burden of PPH mortality and morbidity is concentrated in low‐resource settings [2, 3]. PPH is traditionally defined as estimated blood loss (EBL) ≥ 500 mL from the genital tract during the puerperium and complicates approximately 6% of births annually [4]. Severe PPH (EBL ≥ 1000 mL) complicates one to 2% of births [5]. Due to the subjective nature of estimating blood loss in labour, the definition of PPH has been updated and now includes signs of clinical shock regardless of the volume of EBL [6].

PPH is difficult to predict and occurs frequently in women without identifiable risk factors [6, 7]. Thus, preventative care with active management of the third stage of labour, including uterotonic agents to promote uterine contraction, is required [8]. There are many uterotonic agents, all with differing effectiveness and maternal side effect profiles.

In 2018, the Cochrane Collaboration published a network meta‐analysis (NMA) evaluating all uterotonics for preventing PPH [9] which found that all uterotonic agents are effective in preventing PPH when compared with placebo or no treatment. Specifically, regarding oxytocin compared to placebo or no intervention, Cochrane found that oxytocin was associated with a significant reduction in the risk of PPH and severe PPH as compared to no intervention. In clinical practice, oxytocin is the most frequently used uterotonic for active third‐stage management due to its proven effectiveness, relatively few maternal side effects, and low cost [10, 11].

In general, systematic reviews (SR) and meta‐analyses (MA) of randomised controlled trials (RCTs) provide the highest level of evidence and certainty of a particular treatment's effect size. However, the results of SRs and MAs are only reliable if the underlying RCTs are trustworthy. There is increasing evidence that data with compromised integrity are included in evidence synthesis within medicine [12, 13, 14, 15], and also in obstetrics and gynaecology [16, 17]. MA with individual participant data (IPD‐MA) allows assessment of the trustworthiness of RCT data. Here, we report an IPD‐MA assessing the effectiveness of oxytocin for preventing PPH.

## 2 Objectives

This IPD and AD‐MA aimed to compare the effectiveness and maternal safety of oxytocin to no intervention for preventing PPH. The MA aimed to pool data from trustworthy RCTs and compare the results with data from RCTs not meeting trustworthiness criteria. By doing so, we will understand how the trustworthiness of the RCTs impacts the effect estimates of oxytocin as compared to no intervention for preventing PPH.

## 3 Methods

This IPD‐MA followed a prospectively registered protocol (PROSPERO: CRD42022348464, accessed from: https://www.crd.york.ac.uk/prospero/). Ethical approval was received from Monash University Human Research Ethics Committee in compliance with the requirements of the National Statement on Ethical Conduct in Human Research, Project ID: 34839.

### 3.1 Search Strategy and Eligibility Criteria

Relevant RCTs from the 2018 Cochrane NMA were included [9]. Using the same inclusion criteria, with the help of an information specialist at Cochrane, we updated the search with RCTs published between May 2018 to May 2023 (Figure S1). All RCTs, published and unpublished, comparing systemic oxytocin to placebo or no intervention for preventing PPH were eligible. No limits were set on the dose of oxytocin, route of administration, or the mode of delivery. No language restrictions were used. Two investigators (AR and MF) independently screened articles, and disagreements were resolved by a third reviewer (MP).

### 3.2 Data Access

We approached investigators of eligible RCTs to share IPD. Trial investigators' contact details were obtained through the published articles or their institutional websites. IPD‐MA invitations were e‐mailed at least four times if there was no response. Where the corresponding authors' contact details were unavailable or no response was obtained, attempts were made to contact other authors involved in the RCTs, and co‐authors were copied in. If authors were not responding to e‐mails, other contact details were sought from institutional affiliations and social media platforms. Our academic contacts in particular countries were also used to contact the authors and/or their institutions who were not responding to the initial enquiries. Journal editors were contacted as a last resort for some studies.

RCT investigators who agreed to partake in this study supplied de‐identified IPD. Data was requested for all women randomised, even if excluded from original trial analyses.

### 3.3 Quality Assessment

#### 3.3.1 Studies That Shared IPD

The received data were harmonised and recoded to the pre‐defined IPD‐MA definitions. They were examined for missing data, error, internal consistency, consistency with the publication, and pattern of treatment allocation and data presentation, where possible [18]. Identified issues were communicated with RCT investigators for a solution.

#### 3.3.2 Studies That Did Not Share IPD

The Trustworthiness in RAndomised Clinical Trials (TRACT) data integrity tool [19] was used to assess the trustworthiness of studies that did not provide IPD. This checklist surveys seven domains, including governance, author group, plausibility of intervention, time frame, dropout rates, baseline characteristics, and outcomes; it aims to make an objective assessment regarding a trial's trustworthiness. If needed, we contacted the authors for clarification.

#### 3.3.3 Risk of Bias Assessment

The risk of bias (RoB) was evaluated by one reviewer (AR) for all studies using the Cochrane RoB‐2 tool [20]. The RCTs were categorised into ‘low’, ‘some concerns’ and ‘high’ risk of data integrity concerns. In cases where information was incomplete, clarification was sought from the trial authors. The RoB‐2 scores were then compared with those from the 2018 Cochrane NMA for consistency. The GRADE tool was applied by one reviewer (AR), with results compared to the 2018 Cochrane NMA.

### 3.4 Outcomes

Primary outcomes were PPH ≥ 500 mL and severe PPH ≥ 1000 mL. Secondary outcomes were EBL (mL), duration of the third stage of labour (minutes), need for additional uterotonics, blood transfusion, manual removal of placenta, admission to intensive care unit (ICU), headache, nausea, vomiting, shivering, diarrhoea, and pyrexia (Figure S2).

### 3.5 Data Synthesis

For each outcome, an intention‐to‐treat analysis was performed using all available data comparing oxytocin and placebo or no intervention. In this IPD‐MA, placebo or no intervention was considered the reference group for all outcomes.

Our primary analysis was a two‐stage MA to synthesise the IPD. If we were unable to use a two‐stage approach due to the occurrence of rare events, then a one‐stage approach was used. In the first step of the two‐stage method, we compared outcomes between oxytocin and placebo or no intervention for each included study. For binary outcomes, odds ratios (ORs) were calculated along with 95% confidence intervals (CIs) using logistic regression. In the second step, relative estimates were combined using random‐effects models (restricted maximum likelihood estimator with Hartung‐Knapp‐Sidik‐Jonkman variance correction) [21]. We tested treatment‐covariate interactions for PPH using interaction terms between treatment and potential effect modifiers. Only within‐trial interaction was considered to avoid ecological bias.

All variables besides the identification variable were checked for missing values and entries outside the expected ranges. Variables that were missing > 0.01% of observations were analysed separately for each dataset using the patterns chart of missing data. In the event of missing values for covariates or potential effect modifiers in any RCT, multiple imputations using chained equations (10 imputed datasets) were performed within the RCT before the MA [22].

AD‐MA using the same random‐effects model was performed to assess the risk of data unavailability bias of the IPD‐MA. The treatment effect using IPD and AD of studies that met trustworthiness criteria was assessed. Finally, the treatment effect of the RCTs that did not meet trustworthiness criteria was assessed.

We performed post hoc subgroup analyses for placebo‐controlled RCTs versus open‐label RCTs comparing oxytocin versus no intervention. We also performed post hoc subgroup analyses for the dose of oxytocin: 10 international units (IU) with < 10 IU, comparing oxytocin versus no intervention for the primary outcomes.

Stata/SE version 18.0, provided by StataCorp in College Station, Texas, USA, was used for statistical analysis. The ipdmetan, meqrlogit, and meta commands within Stata were used for conducting the MA.

## 4 Results

### 4.1 Study Selection and Participants

**FIGURE 1:**
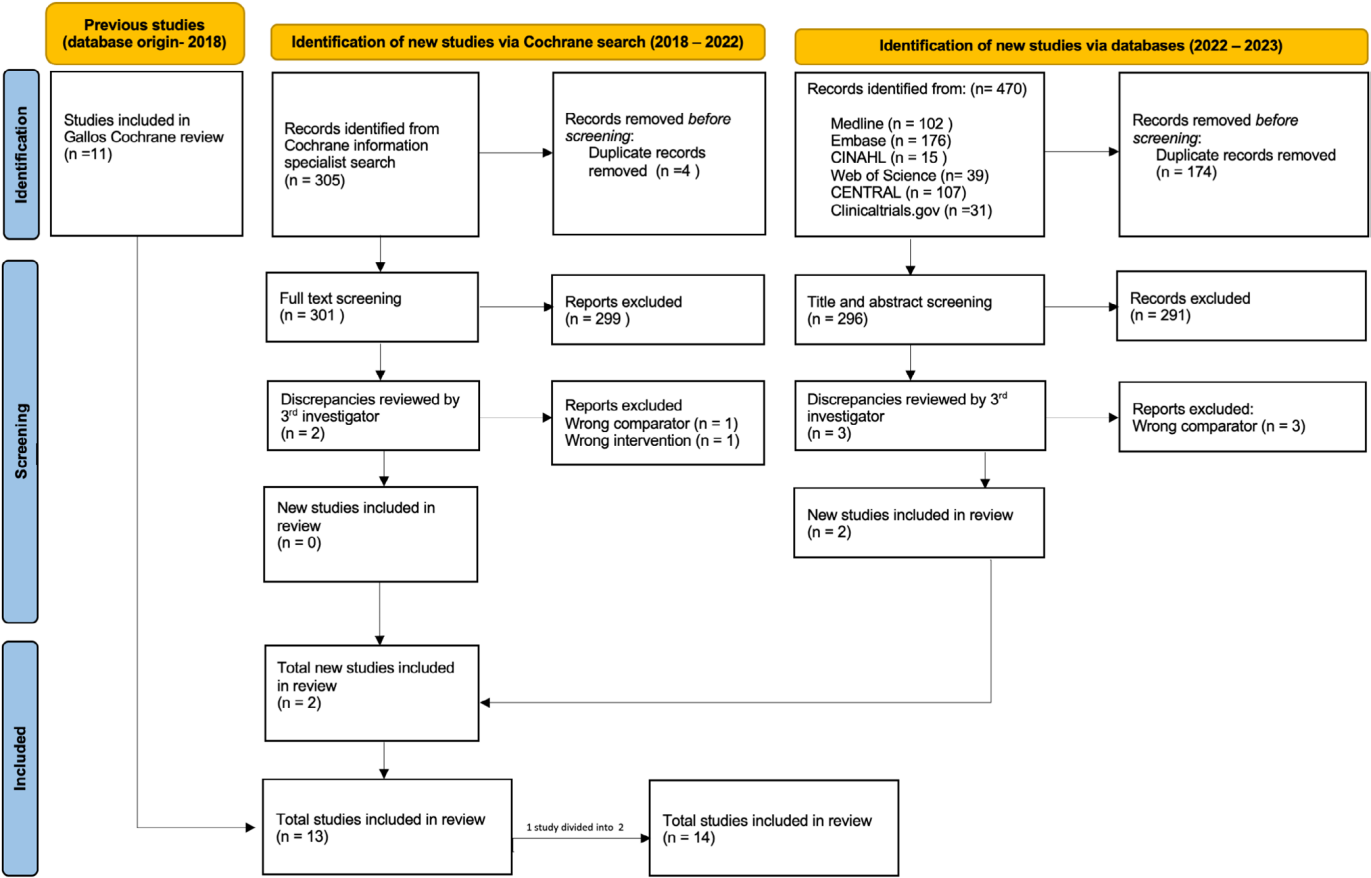
Trial identification (PRISMA‐IPD flow diagram). PRISMA, Preferred Reporting Items for Systematic reviews and Meta‐Analyses.

We screened 196 RCTs from the Cochrane 2018 NMA, which compared uterotonics for preventing PPH [9]. Eleven RCTs comparing oxytocin to placebo or no intervention were eligible for inclusion (Table S1). An additional systematic search, conducted by the Cochrane information specialist, identified 305 unique references; however, after abstract and full‐text screening, none were eligible for inclusion. A further 470 studies were retrieved from databases, with screening identifying two additional eligible RCTs, bringing the total to 13 (see PRISMA‐IPD flow diagram, Figure 1). One multicentre RCT [23] was conducted in two countries (Assiut, Egypt and Eastern Cape, South Africa) and was reported as two separate RCTs in one publication. We considered these as two separate RCTs, thus increasing the total number of studies from 13 to 14.

Of the 14 RCT authors, two did not respond to our invitation [24, 25]. Of the 12 who responded, four agreed to participate, while eight declined. The primary reasons for declining were the unavailability of IPD, either due to the inability to retrieve the data (*n* = 3) or because the original authors had retired (*n* = 2) or were deceased (*n* = 2). Other reasons for declining were being too busy to participate (*n* = 1). A detailed summary of the included RCTs, author responses, and reasons for non‐participation is provided in Table 1.

**TABLE 1:** Responses of the trialists to the invitation to participate and trustworthiness classification.

Author	Year	Country	Route and dose of oxytocin	Comparator	# Pts	Outcome of invitation	Explanation	TRACT
Ilancheran [1]	1990	Singapore	IV^a^	No intervention	10	Declined	Authors deceased	Low risk
Poeschmann [2]	1991	Netherlands	5 IU IM	Placebo	52	Declined	Authors deceased	Low risk
de Groot [3]	1996	Netherlands	5 IU IM	Placebo	221	Declined	Unable to locate data	Low risk
Nordstrom [4]	1997	Sweden	10 IU IV	Placebo	1000	Declined	Authors deceased/retired	Low risk
Bader [5]	2000	Germany	3 IU IV	No intervention	120	Declined	Unable to locate data	Low risk
Benchimol [6]	2001	France	2.5 IU IV	No intervention	382	Declined	Unable to locate data	Low risk
Jerbi [7]	2007	Tunisia	5 IU IV	No intervention	130	No response		High risk
Abdel‐Aleem [8]	2010	Egypt	10 IU IM	No intervention	951	Declined	Too busy to participate	High risk
Hofmeyr [8]	2010	South Africa	10 IU IM	No intervention	1013	Accepted	IPD received	Low risk
Jangsten [9]	2011	Sweden	10 IU IV	Placebo	1631	Declined	Authors retired	Low risk
Al‐Sawaf [10]	2013	Egypt	5 IU IM	No intervention	76	No response		High risk
Rosseland [11]	2013	Norway	5 IU IV	Placebo	51	Accepted	IPD received	Low risk
Stanton [12]	2013	Ghana	10 IU IM	No intervention	1569	Accepted	IPD received	Low risk
Jans [13]	2016	Netherlands	5 IU IM	No intervention	1686	Accepted	IPD received	Low risk

Abbreviations: IM, intramuscular; IU, international units; IV, intravenous; Pt, patients; TRACT, Trustworthiness in RAndomised Controlled Trials assessment.

^a^ Dosage of oxytocin is not available in trial manuscript.

### 4.2 Study Characteristics

Of the four studies that provided IPD, three studies provided complete IPD [26, 27, 28]. One paper reported two separate RCTs conducted in different trial centres [23], the lead trialist in one centre declined participation, while the lead trialist from the other trial centre accepted our invitation and provided IPD (Table 1).

Data veracity of the four IPD sets was tested using a recently published IPD integrity tool [18] and all four were included in our MA [23, 26, 27, 28]. Of these, one study (*n* = 51) [26] used a placebo and the other three (*n* = 4203) [23, 27, 28] had no intervention as the control. Route and dose of oxytocin also varied: two studies administered 10 IU intramuscularly (IM) [23, 27], one administered 5 IU IM [28] and one administered 5 IU intravenously (IV) [26].

### 4.3 Trustworthiness Assessment

Of 10 RCTs that did not provide raw data, seven were regarded as low risk of integrity concerns. Three RCTs were considered high risk for integrity concerns [23, 24, 25] due to previous publication retractions [29], missing trial registration [25, 30, 31] and absent research ethics [24] (Table S5), and thus did not meet the trustworthiness criteria. For Abdel‐Aleem et al., our trustworthiness concerns only related to the Egyptian part of the study [23].

### 4.4 Risk of Bias in Included Studies

All the RCTs were identified as having ‘some concerns’ (*n* = 7) or ‘high risk’ (*n* = 6) of bias (Figures S3 and S4). This is predominantly due to the lack of prospective study registration, as most of the studies were conducted before the 2010 trial registration mandate and because in most RCTs, assessors and patients were not blinded to the treatment allocation.

### 4.5 Descriptive Analysis of Participants

In total, 4304 participants were randomised to prophylactic oxytocin (*n* = 2223; 51.7%) and placebo or no intervention (*n* = 2081; 48.4%). The mean maternal age was 28.5 years for oxytocin and 28.4 years for placebo or no intervention. Parity was similar between groups; 17.7% of patients were nulliparous in the oxytocin arm and 19.7% in the control arm (Table S2).

### 4.6 Synthesis of Results

#### 4.6.1 Primary Outcomes: IPD‐MA

**FIGURE 2:**
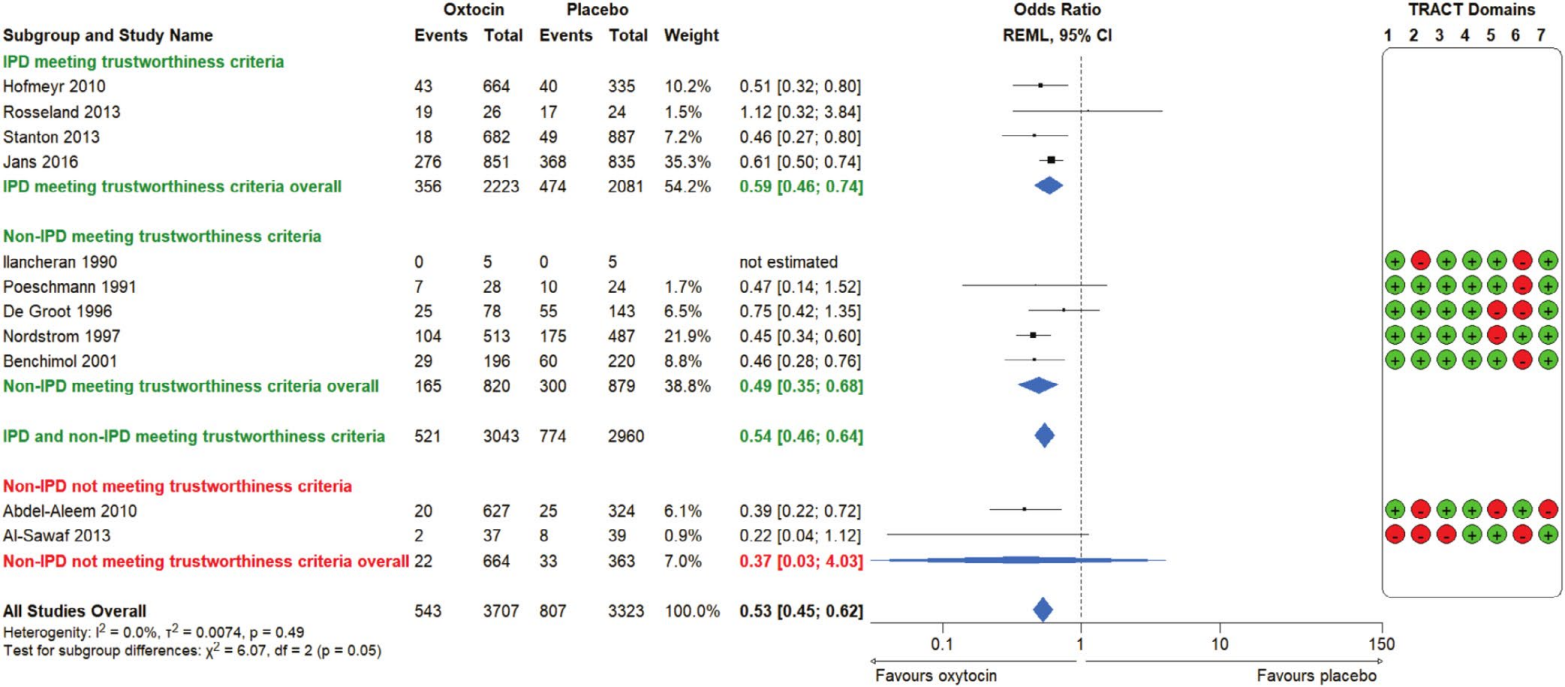
Forest plot comparison and integration of IPD‐MA and aggregate data MA of RCTs meeting or not meeting trustworthiness criteria: Oxytocin compared to placebo or no intervention for the outcome PPH ≥ 500 mL. IPD, individual participant data; REML, restricted maximum likelihood (overall treatment effect estimation); TRACT, Trustworthiness in RAndomised Controlled Trials.

**FIGURE 3:**
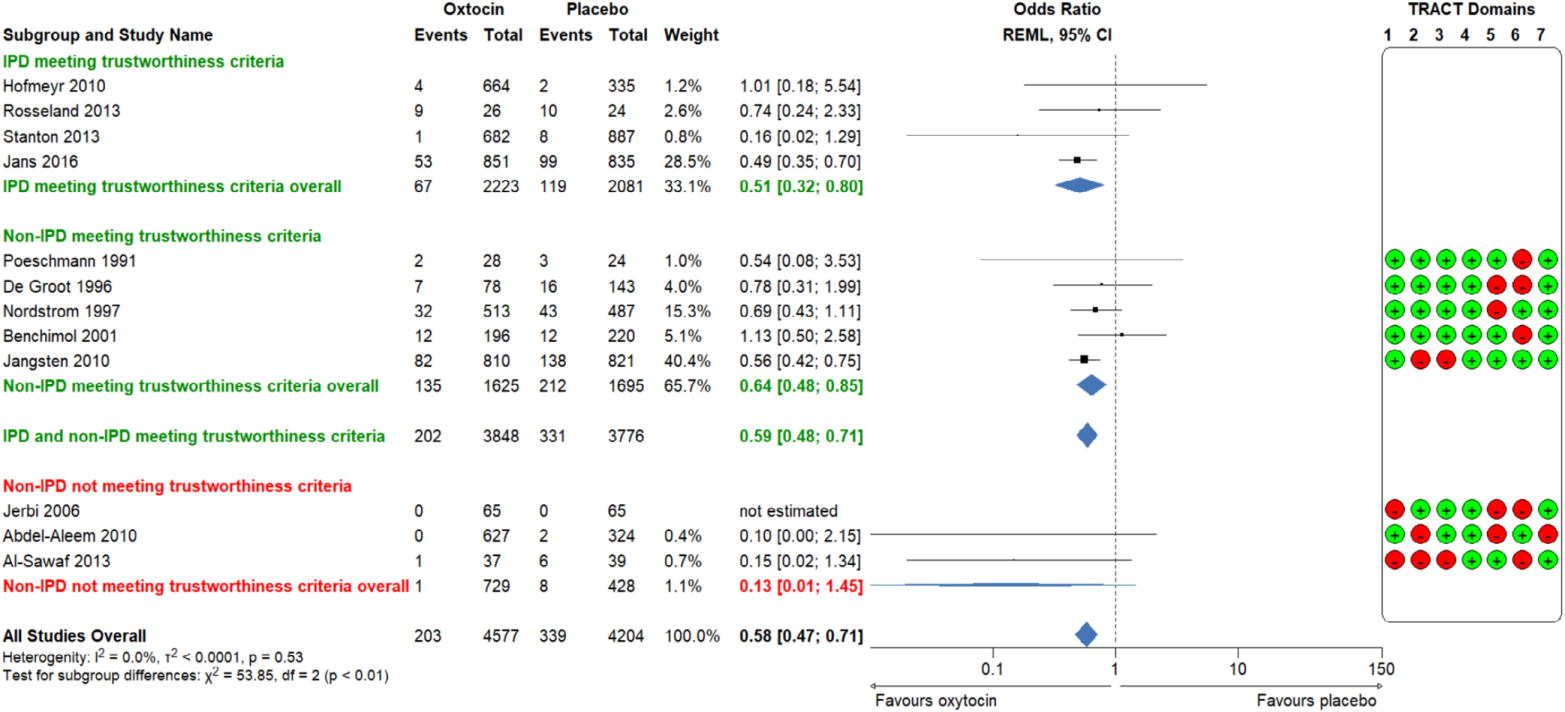
Forest plot comparison and integration of IPD‐MA and aggregate data MA of RCTs meeting or not meeting trustworthiness criteria: Oxytocin compared to placebo or no intervention for the outcome PPH ≥ 1000 mL. IPD, individual participant data; REML, restricted maximum likelihood (overall treatment effect estimation); TRACT, Trustworthiness in RAndomised Controlled Trials.

Oxytocin use was associated with a significant decrease in the rate of PPH ≥ 500 mL and PPH ≥ 1000 mL as compared with placebo/no intervention (PPH ≥ 500 mL: 4 RCTs, *n* = 4304, 16.0% vs. 22.8%, aOR 0.59; 95% CI 0.46 to 0.74; *p* = 0.514; Figure 2. PPH ≥ 1000 mL: 4 RCTs, *n* = 4304, 3.0% vs. 5.7%, aOR 0.51; 95% CI 0.32 to 0.80; *p* = 0.835; Figure 3).

Oxytocin use was associated with a significant decrease in the average EBL (4 RCTs, *n* = 2083, mean difference (MD) 56.54 mL, 95% CI −98.52 to −14.55; Table S3), and a modest decrease in the duration of the third stage by 11 s (3 RCTs, *n* = 2033, 95% CI −0.77 to 0.39; Table S3) as compared with placebo/no intervention. Oxytocin use was associated with a non‐significant decrease in additional uterotonic use (2 RCTs, *n* = 171, risk ratio (RR) 0.74, 95% CI 0.21 to 2.66; Table S3), and blood transfusion requirement (2 RCTs, *n* = 31, RR 0.96, 95% CI 0.24 to 3.93; Table S3) as compared with placebo/no intervention.

Analysis of maternal adverse effects was limited due to small sample sizes. Two studies reported maternal headache; however, the risk was uncertain given the wide CIs (*n* = 96; RR 6.5, 95% CI 0.35 to 119; Table S3).

### 4.7 Integrated MA: IPD and AD

Analysis of studies meeting trustworthiness criteria (IPD and AD) showed that oxytocin use was associated with a significantly decreased risk of PPH ≥ 500 mL (9 RCTs, *n* = 6003, aOR 0.54; 95% CI 0.46 to 0.64; *p* = 0.489, Figure 2). Two RCTs that did not meet trustworthiness criteria showed that oxytocin was associated with a non‐significant decreased risk of PPH ≥ 500 mL; however, the risk was uncertain given the wide CI (2 RCTs, *n* = 1027, aOR 0.37; 95% CI 0.03 to 4.03; *p* = 0.514, Figure 2). Analysis of all data suggested that oxytocin use was associated with a significantly decreased risk of PPH ≥ 500 mL (11 RCTs, *n* = 7030, aOR 0.53; 95% CI 0.45 to 0.62; *p* = 0.489, Figure 2).

Regarding severe PPH ≥ 1000 mL, analysis of studies meeting trustworthiness criteria (IPD and AD) showed that oxytocin use was associated with a significant decrease in the risk of PPH ≥ 1000 mL (9 RCTs, *n* = 7624, aOR 0.59; 95% CI 0.48 to 0.71; *p* = 0.612, Figure 3). Three RCTs did not meet our trustworthiness criteria; the risk of PPH ≥ 1000 mL was unable to be accurately estimated given the wide CI (3 RCTs, *n* = 1157, aOR 0.13; 95% CI 0.01 to 1.45; *p* = 0.835, Figure 3). Analysis of all data suggested that oxytocin significantly decreased the risk of PPH ≥ 1000 mL (12 RCTs, *n* = 8781, aOR 0.58; 95% CI 0.47, 0.71; *p* = 0.530, Figure 3).

Regarding EBL, studies meeting trustworthiness criteria (IPD and AD) showed that oxytocin use decreased average blood loss by 76 mL (10 RCTs, *n* = 7738, MD −75.9, 95% CI −112.97 to −38.87; Table S4). Analysis of all data suggested that oxytocin use significantly decreased EBL by 81 mL (11 RCTs, *n* = 7814, MD −80.89; 95% CI −115.21 to −46.57, Table S4).

Regarding the length of the third stage of labour, studies meeting trustworthiness criteria (IPD and AD) showed that oxytocin use was associated with a modest reduction in the length of the third stage of labour (7 RCTs, *n* = 6231, MD −0.54 min, 95% CI −1.25 to 0.16, Table S4). Analysis of all RCTs suggested that oxytocin decreased the length of the third stage by 2 min (8 RCTs, *n* = 6361, MD −1.82 min; 95% CI −4.59 to 0.96, Table S4).

### 4.8 Post Hoc Subgroup Analysis

#### 4.8.1 Placebo‐Controlled RCTs Versus Open‐Label RCTs Comparing Oxytocin to no Intervention

Post hoc subgroup analysis for placebo‐controlled RCTs versus open‐label RCTs comparing oxytocin to no intervention included only trustworthy data. Of 11 trustworthy RCTs, 5 were placebo‐controlled and 6 were open label. For the outcome PPH ≥ 500 mL, MA of placebo‐controlled RCTs showed that oxytocin was associated with a non‐significant decreased risk of PPH (4 RCTs, 1323 participants; OR: 0.57, 95% CI 0.32 to 1.00, Table S6). MA of open‐label RCTs showed that oxytocin was associated with a significant decreased risk of PPH (5 RCTs, 4860 participants; OR 0.56, 95% CI 0.46 to 0.69, Table S6).

For PPH ≥ 1000 mL, analysis of placebo‐controlled RCTs showed that oxytocin was associated with a significant decrease in the risk of PPH (5 RCTs, 2954 participants; OR 0.61, 95% CI 0.51 to 0.71, Table S7). Similarly, analysis of open‐label RCTs showed that oxytocin was associated with a non‐significant decreased risk of PPH (4 RCTs, 4670 participants; OR 0.62, 95% CI 0.23 to 1.68, Table S7).

There was no significant difference between the placebo‐controlled and open‐label subgroups for PPH ≥ 500 mL(*p* = 0.960, Table S6) or PPH ≥ 1000 mL (*p* = 0.927, Table S7).

#### 4.8.2 RCTs With Oxytocin 10 IU Versus RCTs With Oxytocin < 10 IU Comparing Oxytocin to No Intervention

Of 11 trustworthy RCTs, nine reported the primary outcomes PPH ≥ 500 mL and ≥ 1000 mL. Four of these RCTs used oxytocin 10 IU and five RCTs used doses < 10 IU (oxytocin = 5 IU (*n* = 2); oxytocin = 2.5 IU (*n* = 1)).

For the outcome PPH ≥ 500 mL, analysis of trustworthy data showed that both subgroups, RCTs using oxytocin 10 IU and RCTs using oxytocin < 10 IU, were associated with a significantly decreased risk of PPH (oxytocin 10 IU: 3 RCTs, 3569 participants, OR 0.47, 95% CI 0.40 to 0.54; oxytocin < 10 IU: 5 RCTs, 2425 participants, OR 0.60, 95% CI 0.49 to 0.74, Table S8). There was a significant difference between these groups; oxytocin 10 IU was associated with a significantly decreased risk of PPH ≥ 500 mL as compared with oxytocin < 10 IU (p value between subgroups = 0.002).

For the outcome PPH ≥ 1000 mL, subgroup analysis of RCTs using oxytocin 10 IU showed that oxytocin was associated with a significantly decreased risk of PPH (4 RCTs, 5199 participants, OR 0.59, 95% CI 0.41 to 0.80, Table S9). Analysis of RCTs using oxytocin < 10 IU showed that oxytocin was associated with a non‐significant decreased risk of PPH (5 RCTs, 2425 participants, OR 0.65, 95% CI 0.42 to 1.03, Table S9). There was no significant difference between the oxytocin 10 IU and oxytocin < 10 IU subgroups for PPH ≥ 1000 mL (*p* = 0.578).

## 5 Discussion

### 5.1 Main Findings

Jointly considering the results of IPD‐MA and AD‐MA of all RCTs meeting trustworthiness criteria, we found that oxytocin administered in the third stage of labour significantly decreases the risk of PPH and severe PPH. Three RCTs did not meet our pre‐defined trustworthiness criteria; meta‐analysis of these RCTs was difficult to interpret given the overall low PPH event rates and wide CIs.

### 5.2 Strengths and Limitations

One of the major strengths of this study was the large sample size, with a total of four RCTs, totalling 4304 participants; 2223 (51.7%) participants received oxytocin and 2081 (48.4%) received no intervention or placebo. Data were received from three large trials, increasing the external validity as our findings were unlikely to be driven by one RCT.

The IPD‐MA study design provided the platform for a collaborative process between the primary research team and trial investigators. This allowed for accurate and reliable investigation and validation of the raw data [13]. IPD‐MA pools trial data, providing higher statistical power and more accurate treatment effect calculations. The data sets were coded for standardisation, allowing for more uniform analysis and true comparison between the studies.

The RCT trustworthiness assessment is both a strength and a potential limitation of our study. The trustworthiness assessment of IPD was performed through data replication [18], and for studies that did not contribute IPD, the trial publication was assessed with the TRACT tool [19]. Including an assessment of data trustworthiness is a strength as this prevents data that do not meet trustworthiness criteria from being included in evidence synthesis.

However, performing a trustworthiness assessment is a relatively new concept, and there is an inherent degree of subjectivity. While the TRACT checklist [19] and other similar tools in this field [32, 33] are relatively new, increased experience in identifying trustworthiness issues will help improve standardisation [34]. Furthermore, to decrease the mis‐categorisation of RCTs, multiple investigators discussed and agreed upon the final TRACT assessment [35].

Furthermore, the trustworthiness assessment may be biased towards older studies. Firstly, there is decreased data availability for older studies. Secondly, the standards for reporting have developed considerably over time [36]; consequently, the trustworthiness criteria of older studies are not reported with enough detail to allow proper assessment.

Our study has several limitations. IPD was only available for four of the 14 RCTs. Many of the identified RCTs were conducted many years ago; authors of seven studies had either passed away, retired, or were unable to locate their data. Due to limited data, secondary outcomes (including manual placenta removal, ICU admission, vomiting and pyrexia) could not be assessed, and subgroup analyses (mode of birth, risk of PPH, health care setting and dosage, regimen and route of oxytocin administration) were unable to be performed.

Regarding the IPD, patient baseline characteristics were similar between oxytocin and placebo/no intervention groups; however, less than 20% of patients in the oxytocin and placebo/no intervention groups were nulliparous. Similarly, the prepartum Hb levels, while being similar between groups, were low. Therefore, results from the IPD‐MA analysis may be less generalisable to nulliparous and non‐anaemic women.

Limited data posed a challenge for both IPD and AD‐MA. IPD‐MA secondary outcomes such as maternal headache had very few events; this resulted in effect estimates with uncertain CIs. Similarly, it was difficult to quantify the impact of data not meeting trustworthiness criteria on the AD‐MA. Two RCTs not meeting trustworthiness criteria reported the outcome PPH of ≥ 500 mL and three reported PPH of ≥ 1000 mL. Furthermore, these few RCTs reported comparatively very few PPH events as compared with the trustworthy data. Therefore, MA of RCTs not meeting trustworthiness criteria resulted in effect estimates with incredibly wide CIs, rendering meaningful interpretation difficult.

### 5.3 Interpretation

The 2018 Cochrane NMA [9] reported a 39% reduction in PPH ≥ 500 mL with oxytocin use when compared to placebo or no intervention (OR 0.61, 95% CI 0.52 to 0.71). Results from IPD‐MA show that oxytocin is associated with a significant risk reduction as compared with placebo/no intervention. For severe PPH ≥ 1000 mL, both the Cochrane NMA and our IPD‐MA showed that oxytocin is associated with a significant risk reduction as compared with placebo/no intervention.

Our results confirm the long‐established hypothesis that oxytocin is more effective than no intervention for reducing PPH. This IPD and AD‐MA was worthwhile as it is the first IPD‐MA to assess oxytocin versus no intervention for PPH prevention and to consider the trustworthiness of these studies.

Including only high‐quality and trustworthy data in meta‐analysis are key to elucidating the true treatment effect size. A recent IPD‐MA [37] with IPD of RCTs assessing tranexamic acid for postpartum bleeding limited the inclusion criteria to RCTs with a sample size above 500 patients; this included only five RCTs and excluded more than 30 smaller RCTs. Indeed, the effect of tranexamic acid was, although still there, much lower than estimated in the previous meta‐analysis of AD [38].

When the 2018 Cochrane NMA [9] was published, no screening tool was applied to ensure the integrity of the included RCTs. Given the increasing evidence of compromised data integrity within women's health [16, 39, 40] and the high‐impact nature of the Cochrane NMA informing global guideline developmen,t [41, 42, 43, 44] the RCTs comparing uterotonics must be interrogated.

The 2018 Cochrane NMA concluded that the highest ranked uterotonics were ergometrine plus oxytocin, misoprostol plus oxytocin, and carbetocin alone. However, since this NMA was published, many included RCTs have had expressions of concern published [45, 46, 47, 48] and many have concerning features, including many RCTs conducted after 2010 that were not registered [49, 50, 51]. The NMA comparing uterotonic agents for preventing PPH should be repeated after exclusion of trials that do not meet pre‐defined trustworthiness criteria.

## 6 Conclusion

Analysis of trustworthy data confirms that oxytocin significantly reduces the risk of PPH and severe PPH compared to no intervention and is associated with improved maternal safety outcomes. Twenty‐one percent of RCTs did not meet our pre‐defined trustworthiness criteria, elucidating the importance of integrity assessment in evidence synthesis.

## Author Contributions

A.R. and M.F. managed the project and coordinated the collaborative process, taking primary responsibility for data collection, verification of individual patient data, data synthesis, manuscript drafting, editing and finalisation. B.W.J.M., W.L., C.A.W. and M.F. formulated the research concept and supervised every stage of the study's implementation. M.F., B.W.J.M. and A.W. provided clinical and editorial oversight. L.S.A. performed statistical analyses. W.L. provided statistical oversight. M.F. and M.P. assisted with the screening of studies, risk of bias screening and Trustworthiness in RAndomised Controlled Trials screening. All authors were involved in the decision to submit the manuscript. All contributing trial investigators supplied data and answered questions about their trials. They also had opportunities to comment on the initial scope, draft protocol and manuscript.

## Disclosure

This study is supported by two NHMRC Investigator Grants (GNT1176437 for B.W.J.M. and GNT2016729 for W.L.). These funding sources had no role in the design, execution, analyses, or data interpretation for this research. M.F. and M.P. are supported by a Research Training Stipend provided by the Australian Government. B.W.J.M. reports consultancy, travel support, and research funding from Merck and consultancy for Organon and Norgine. B.W.J.M. holds stock from ObsEva.

## Ethics Statement

Ethical approval was received from Monash University Human Research Ethics Committee in compliance with the requirements of the National Statement on Ethical Conduct in Human Research, Project ID: 34839, granted on 13/7/2022.

## Conflicts of Interest

MF and MP are supported by a Research Training Stipend, provided by the Australian Government. BWM declared grants from NHMRC, personal fees from ObsEva, personal fees from Merck, personal fees from Guerbet, other from Guerbet, and grants from Merck, outside the submitted work. WL declared a grant from NHMRC that supports this work and received research grant funds from the Norman Beischer Medical Research Foundation which was unrelated to this work.

## Supporting information


Appendix S1.

## Data Availability

The data that support the findings of this study are available on request from the corresponding author. The data are not publicly available privacy or ethical restrictions. The protocol, statistical analysis plan and codebook are available on request. The trial investigators who shared individual participant data for the purposes of the meta‐analysis retain ownership of their trial data and any requests for access to individual participant data should be made directly to them.
