# Supplementary material for: The Effectiveness of Oxytocin for Preventing Postpartum Haemorrhage: An Individual Participant Data Meta‐Analysis
Source: BJOG. 2025 Jul 16;133(1):24–33. doi: 10.1111/1471-0528.18279 (PMC12676194; doi:10.1111/1471-0528.18279)
Supplement: Supplementary file 1 — Appendix S1. [file BJO-133-24-s001.docx]

**The Effectiveness of Oxytocin for the Prevention of Postpartum Haemorrhage: an Individual Participant Data Meta-Analysis: Supplementary Figures and Tables.**

Figures:

**S1.** Search strategy used to identify studies comparing oxytocin with placebo or no intervention.

**S2.** Definition of outcomes and potential effect modifiers.

**S3.** Risk of bias 2 (RoB-2) assessment of included trials in the intention-to-treat population.

**S4.** RoB-2 scoring domains.

Tables:

**S1.** Characteristics of trials included in the individual participant meta-analysis (IPD-MA) comparing oxytocin with placebo or no intervention.

**S2.** Baseline Characteristics of participants included in the IPD-MA.

**S3.** IPD-MA comparing oxytocin with placebo or no intervention: secondary outcomes.

**S4.** Integrated IPD and aggregate data MA comparing oxytocin with placebo or no intervention: secondary outcomes.

**S5.** Application of TRACT tool to included studies that did not contribute IPD.

**S6:** Post-hoc subgroup analysis: oxytocin vs placebo for the prevention of PPH ≥ 500 mL; oxytocin vs no intervention for the prevention of PPH ≥ 500mL.

**S7:** Post-hoc subgroup analysis: oxytocin vs placebo for the prevention of PPH ≥ 1000 mL; oxytocin vs no intervention for the prevention of PPH ≥ 1000mL.

**S8:** Post-hoc sensitivity analysis: oxytocin 10IU vs placebo/no intervention for the prevention of PPH ≥ 500 mL; oxytocin <10IU vs placebo/no intervention for the prevention of PPH ≥ 500 mL.

**S9:** Post-hoc sensitivity analysis: oxytocin 10IU vs placebo/no intervention for the prevention of PPH ≥ 1000 mL; oxytocin <10IU vs placebo/no intervention for the prevention of PPH ≥ 1000 mL.


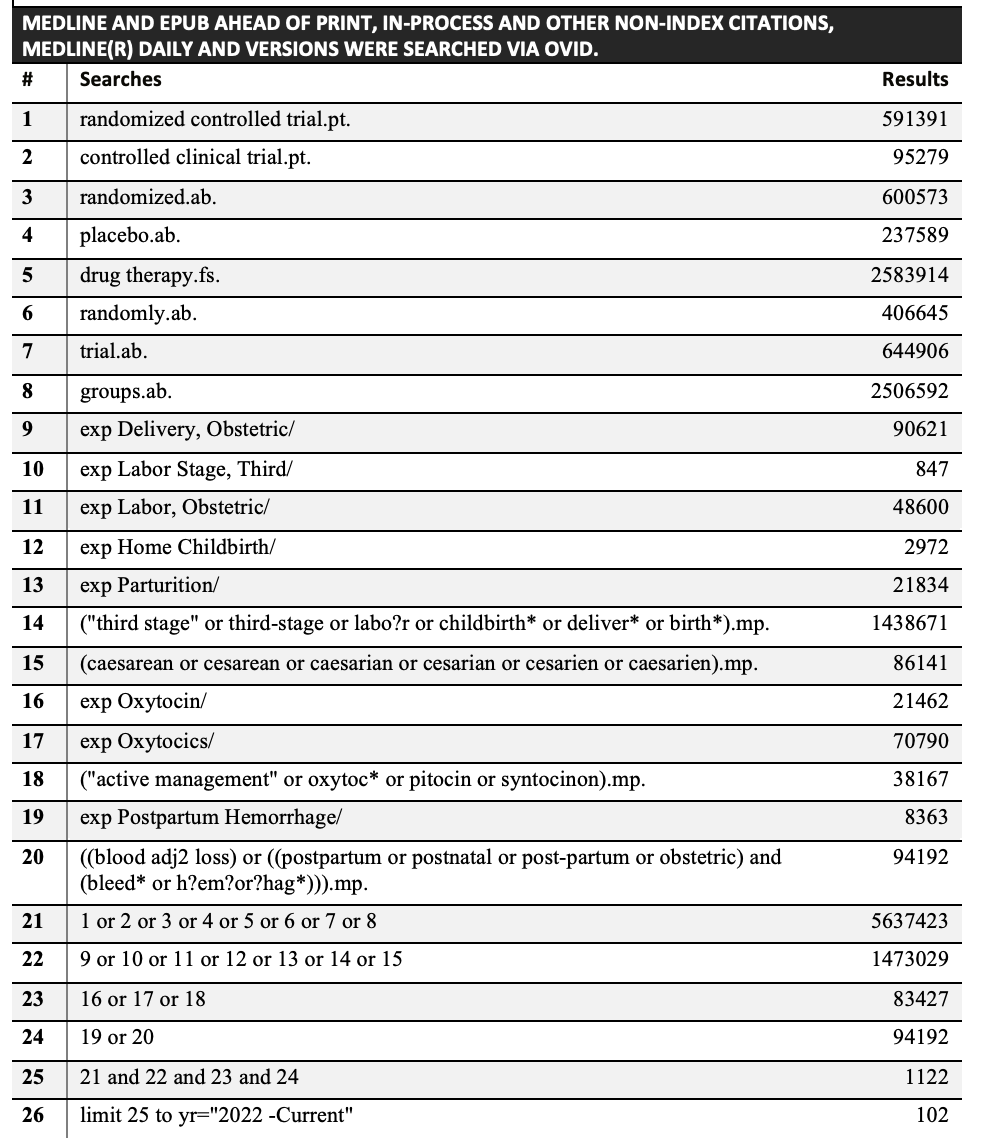


**Figure S1.** Search strategy used to identify studies comparing oxytocin with placebo or no intervention.

**Primary outcomes:**

PPH ≥ 500 mL

PPH ≥ 1000 mL

**Secondary outcomes:**

Outcomes related to blood loss:

Estimated blood loss (EBL; mL)

Need for additional uterotonics

Length of third stage of labour (minutes)

Blood transfusion requirement

Manual removal of placenta*

Admission to intensive care unit*

Outcomes related to maternal side effects:

Headache

Nausea*

Vomiting*

Shivering*

Pyrexia*

Diarrhea*

**Potential effect modifiers:**

Maternal age (in years; MA)

Maternal Parity

- Categorized: nullipara; multiparous

Gestational age (in weeks; GA)

**Figure S2.** Definition of outcomes and potential effect modifiers.

PPH: postpartum haemorrhage.

All intended outcomes are listed in the statistical analysis plan.

*Insufficient data available to perform the planned analysis

**Figure S3.** Risk of bias 2 (RoB-2) assessment of included trials in the intention-to-treat population.


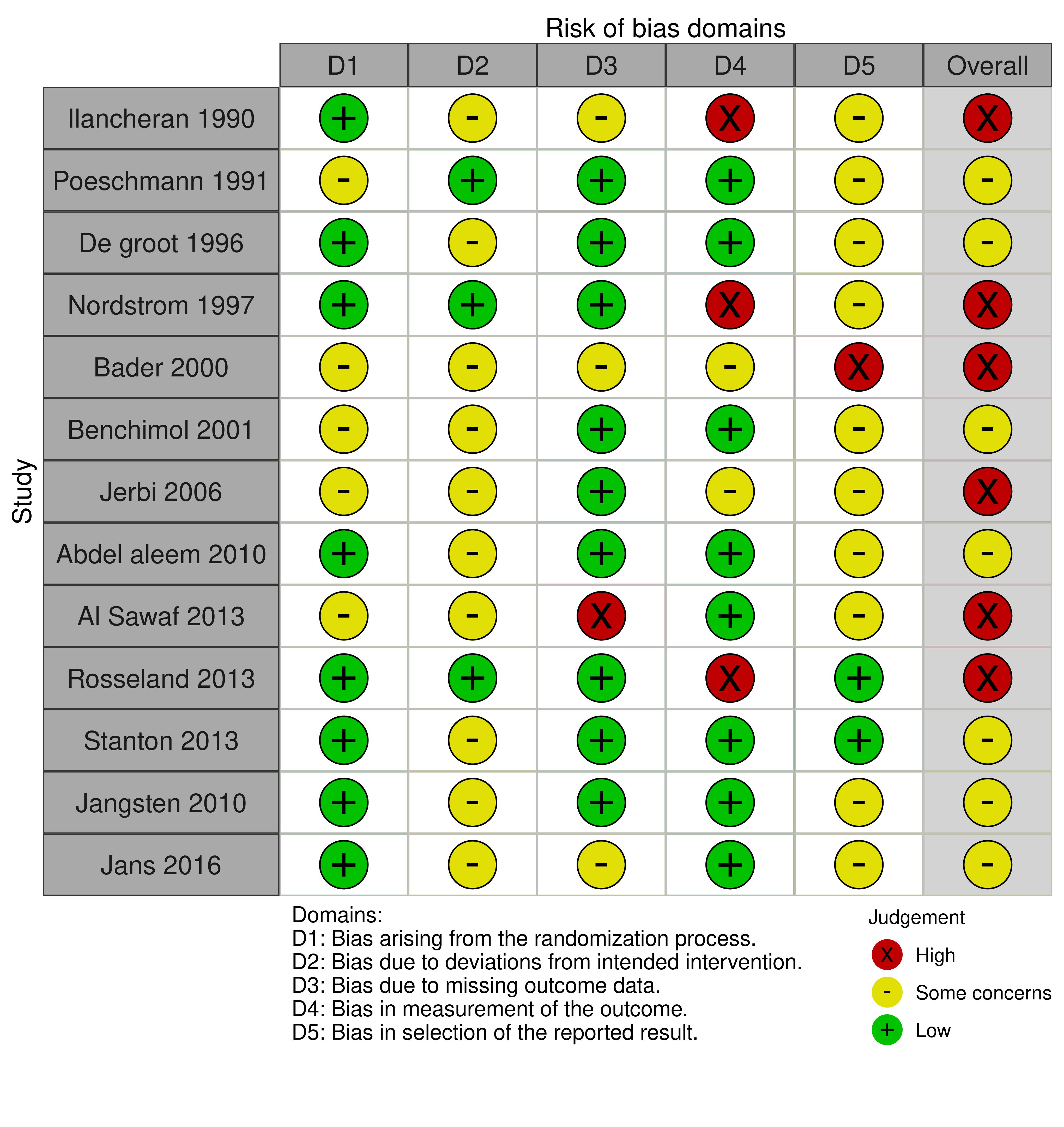


**Figure S4.** RoB-2 scoring domains.

| **#** | **Author** | **Year** | **Country** | **Inclusion criteria** | **# Participants** | **# Cases used** |
| --- | --- | --- | --- | --- | --- | --- |
| 1 | Hofmeyr^(1)^ | 2010 | South Africa | All women with spontaneous vaginal delivery | 999 | 999 |
| 2 | Rosseland^(2)^ | 2013 | Norway | 36+ weeks gestation, age of 18+ with scheduled CS | 51 | 50 |
| 3 | Stanton^(3)^ | 2013 | Ghana | All women birthing at home within the CHO regions | 1,570 | 1,569 |
| 4 | Jans^(4)^ | 2016 | Netherlands | 37+ weeks gestation with midwife led vaginal delivery | 1,686 | 1,686 |

**Table S1.** Characteristics of trials included in the IPD-MA comparing oxytocin with placebo or no intervention.

CS: caesarean section; CHO: community health officer; IPD-MA: Individual participant data meta-analysis.

|  | **Oxytocin** | | **Placebo/no intervention** | | **Overall** | |
| --- | --- | --- | --- | --- | --- | --- |
|  | N= | Mean (SD) | N= | Mean (SD) | N= | Mean (SD) |
| **Age** | 2189 | 28.5 (5.7) | 2051 | 28.4 (5.9) | 4240 | 28.4 (5.8) |
| **Gestational Age (weeks)** | 1398 | 39.1 (2.8) | 1116 | 39.5 (1.8) | 2574 | 39.3 (2.4) |
| **Hb prepartum** | 876 | 7.1 (1.2) | 858 | 7.1 (1.2) | 1734 | 7.1 (1.2) |
| **Height (cm)** | 26 | 169.4 (5.5) | 24 | 168.0 (6.0) | 51 | 168.7 (5.7) |
| **Weight (kg)** | 459 | 79 (30.9) | 240 | 80 (16.1) | 699 | 79 (26.8) |
| **BMI (kg/m^2^)** | 26 | 27.9 (4.0) | 24 | 29.2 (4.3) | 51 | 28.5 (4.2) |
| **Parity***  0 | 233/1316 (17.7) | | 240/1218 (19.7) | | 2060/2534 (18.7) | |
| ≥1 | 1083/1316 (82.3) | | 978/1218 (80.3) | | 474/2534 (81.3) | |
| **Birthweight (grams)** | 698 | 3207 (483) | 377 | 3118 (522) | 1075 | 3198 (506) |

**Table S2.** Baseline Characteristics of participants included in the IPD-MA.

SD: Standard Deviation; Hb: Haemoglobin; IPD-MA: Individual participant data meta-analysis.

* n/N (%)

| **Outcome** | **# Trials** | **# Women** | **Oxytocin**  **n/N (%)** | **Placebo/No intervention**  **n/N (%)** | **Risk Ratio**  **(95% CI) for binary outcomes; MD for continuous outcomes*** | **I^2^**  **(%)** | **P-value** |
| --- | --- | --- | --- | --- | --- | --- | --- |
| **Maternal safety outcomes** | | | | | | |  |
| Need for additional uterotonics | 1 | 2634 | 24/1484 (1.6) | 147/1150 (12.8) | 0.74 (0.21, 2.7) | 0 | - |
| EBL (mL)* | 4 | 4305 | - | - | -*57 (-99, -15) | 0 | 0.023 |
| Blood Transfusion | 1 | 2634 | 16/1481(1.1) | 15/1153 (1.3) | 0.96 (0.24, 3.9) | 0 | - |
| Third stage duration* | 3 | 4215 | - | - | -*0.19 (-0.77, 0.38) | 0 | 0.286 |
| Manual removal of placenta | 0 |  | Insufficient data | | | |  |
| Admission to intensive care unit | 0 |  | Insufficient data | | | |  |
| **Maternal adverse effects** | | | | | | |  |
| Headache | 2 | 96 | 3/49 (6.1) | 0/49 (0.0) | 6.5 (0.35, 119) | NA |  |
| Nausea | 1 |  | Insufficient data | | | |  |
| Vomiting | 1 |  | Insufficient data | | | |  |
| Shivering | 0 |  | Insufficient data | | | |  |
| Pyrexia | 0 |  | Insufficient data | | | |  |
| Diarrhoea | 0 |  | Insufficient data | | | |  |

**Table S3.** IPD-MA comparing oxytocin with placebo or no intervention: secondary outcomes.

MD: mean difference; EBL: estimated blood loss.

One-stage meta-analysis used. Analysis adjusted for maternal age, parity and gestational age.

| **Secondary outcomes** | **# Trials** | **# Women** | **Oxytocin**  **(n= )** | **Placebo/No intervention**  **(n= )** | **Mean Difference**  **(95% CI)** |
| --- | --- | --- | --- | --- | --- |
| **Estimated Blood Loss** | | | | | |
| IPD and non-IPD meeting trustworthiness criteria | 10 | 7738 | 3901 | 3837 | -75.9 (-113.0, -38.9) |
| Non-IPD not meeting trustworthiness criteria | 1 | 76 | 37 | 39 | -123.9 (-174.9, -72.9) |
| All studies | 11 | 7814 | 3938 | 3876 | -80.9 (-115.2, -46.6) |
| **Length of third stage** | | | | | |
| IPD and non-IPD meeting trustworthiness criteria | 7 | 6231 | 3151 | 3080 | -0.54 (-1.25, 0.16) |
| Non-IPD not meeting trustworthiness criteria | 1 | 130 | 65 | 65 | -8.1 (-9.7, -6.5) |
| All studies | 8 | 6361 | 3216 | 3145 | -1.82 (-4.6, 0.96) |

**Table S4.** Integrated IPD and aggregate data MA comparing oxytocin with placebo or no intervention: secondary outcomes.

IPD: individual participant data; MA: meta-analysis

**Table** **S5.** Application of TRACT tool to included studies that did not contribute IPD.

TRACT: Trustworthiness in RAndomised Controlled Trials; IPD: Individual participant data.

| **PPH ≥ 500 mL** | RCTs (n= ) | Pts (n= ) | Crude incidence: oxytocin vs placebo/no intervention: n/N (%) | OR (95% CI) | P-value | P value between subgroup |
| --- | --- | --- | --- | --- | --- | --- |
| **Placebo** | | | | | |  |
| Trustworthy IPD | 1 | 50 | Oxytocin: 19/26 (73.1) | 1.12 (0.32, 3.84) | - |  |
|  |  |  | Placebo: 17/24 (70.8) |  |  |  |
| Trustworthy non-IPD | 3 | 1,273 | Oxytocin: 136/619 (22.0) | 0.53 (0.26, 1.06) | 0.059 |  |
|  |  |  | Placebo: 240/654 (36.7) |  |  |  |
| Trustworthy Overall | 4 | 1,323 | Oxytocin: 155/645 (24.0) | 0.57 (0.32, 1.00) | 0.050 |  |
|  |  |  | Placebo: 257/678 (37.9) |  |  |  |
| **No intervention** | | | | | |  |
| Trustworthy IPD | 3 | 4,254 | Oxytocin: 337/2197 (15.3) | 0.58 (0.43, 0.77) | 0.015 |  |
|  |  |  | No intervention: 457/2057 (22.2) |  |  |  |
| Trustworthy non-IPD | 2 | 426 | Oxytocin: 29/201(14.4) | 0.46 (0.28, 0.76) | - |  |
|  |  |  | No intervention: 60/225 (26.7) |  |  |  |
| Trustworthy Overall | 5 | 4,860 | Oxytocin: 366/2398 (15.3) | 0.56 (0.46, 0.69) | 0.003 | 0.960 |
|  |  |  | No intervention: 517/2282 (22.7) |  |  |  |

**Table S6:** Post-hoc subgroup analysis: oxytocin vs placebo for the prevention of PPH ≥ 500 mL; oxytocin vs no intervention for the prevention of PPH ≥ 500mL.

PPH: Postpartum haemorrhage; RCTs: Randomised controlled trials; Pts: Participants; OR: Odds ratio; IPD: Individual participant data

| **PPH ≥ 1000 mL** | RCTs (n= ) | Pts (n= ) | Crude incidence: oxytocin vs placebo/no intervention: n/N (%) | OR (95% CI) | P-value | P value between subgroup |
| --- | --- | --- | --- | --- | --- | --- |
| **Placebo** | | | | | |  |
| Trustworthy IPD | 1 | 50 | Oxytocin: 19/26 (73.1) | 0.74 (0.24, 2.33) | - |  |
|  |  |  | Placebo: 17/24 (70.8) |  |  |  |
| Trustworthy non-IPD | 4 | 2,904 | Oxytocin: 123/1429 (8.6) | 0.60 (0.49, 0.74) | 0.004 |  |
|  |  |  | Placebo: 200/1475 (13.6) |  |  |  |
| Trustworthy Overall | 5 | 2,954 | Oxytocin: 132/1455 (9.1) | 0.61 (0.51, 0.71) | 0.001 |  |
|  |  |  | Placebo: 210/1499 (14.0) |  |  |  |
| **No intervention** | | | | | |  |
| Trustworthy IPD | 3 | 4,254 | Oxytocin: 58/2197 (2.6) | 0.49 (0.25, 0.99) | 0.049 |  |
|  |  |  | No intervention: 109/2057 (5.3) |  |  |  |
| Trustworthy non-IPD | 1 | 416 | Oxytocin: 12/196 (6.1) | 1.13 (0.50, 2.58) | - |  |
|  |  |  | No intervention: 12/220 (5.5) |  |  |  |
| Trustworthy Overall | 4 | 4,670 | Oxytocin: 70/2393 (2.9) | 0.62 (0.23, 1.68) | 0.226 | 0.927 |
|  |  |  | No intervention: 121/2277 (5.3) |  |  |  |

**Table S7:** Post-hoc subgroup analysis: oxytocin vs placebo for the prevention of PPH ≥ 1000 mL; oxytocin vs no intervention for the prevention of PPH ≥ 1000mL.

PPH: Postpartum haemorrhage; RCTs: Randomised controlled trials; Pts: Participants; OR: Odds ratio; IPD: Individual participant data

| **PPH ≥ 500 mL** | RCTs (n= ) | Pts (n= ) | Crude incidence: oxytocin vs placebo/no intervention: n/N (%) | OR (95% CI) | P value | P value between subgroup |
| --- | --- | --- | --- | --- | --- | --- |
| **Oxytocin 10 IU** |  |  |  |  |  |  |
| Trustworthy IPD | 2 | 2,568 | Oxytocin: 61/1346 (4.5) | 0.49 (0.27, 0.90) | 0.042 |  |
|  |  |  | Placebo/no intervention: 89/1222 (7.3) |  |  |  |
| Trustworthy non-IPD | 1 | 1,000 | Oxytocin: 104/513 (20.3) | 0.45 (0.34, 0.60) | - |  |
|  |  |  | Placebo/no intervention: 175/487 (35.9) |  |  |  |
| Trustworthy Overall | 3 | 3,569 | Oxytocin: 165/1859 (8.9) | 0.47 (0.40, 0.54) | 0.002 |  |
|  |  |  | Placebo/no intervention: 264/1709 (15.4) |  |  |  |
| **Oxytocin < 10IU** |  |  |  |  |  |  |
| Trustworthy IPD | 2 | 1,736 | Oxytocin: 295/877 (33.6) | 0.62 (0.19, 2.07) | 0.124 |  |
|  |  |  | Placebo/no intervention: 385/859 (44.8) |  |  |  |
| Trustworthy non-IPD | 3 | 689 | Oxytocin: 61/302 (20.2) | 0.56 (0.27, 1.15) | 0.074 |  |
|  |  |  | Placebo/no intervention: 125/387 (32.3) |  |  |  |
| Trustworthy Overall | 5 | 2,425 | Oxytocin: 356/1179 (30.2) | 0.60 (0.49, 0.74) | 0.002 | 0.002 |
|  |  |  | Placebo/no intervention: 510/1246 (40.9) |  |  |  |

**Table S8:** Post-hoc sensitivity analysis: oxytocin 10IU vs placebo/no intervention for the prevention of PPH ≥ 500 mL; oxytocin <10IU vs placebo/no intervention for the prevention of PPH ≥ 500 mL.

PPH: Postpartum haemorrhage; RCTs: Randomised controlled trials; Pts: Participants; OR: Odds ratio; IPD: Individual participant data; IU: International units.

| **PPH ≥ 1000 mL** | RCTs (n= ) | Pts (n= ) | Crude incidence: oxytocin vs placebo/no intervention: n/N (%) | OR (95% CI) | P value | P value between subgroup |
| --- | --- | --- | --- | --- | --- | --- |
| **Oxytocin 10 IU** | | | | | |  |
| Trustworthy IPD | 2 | 2,568 | Oxytocin: 5/1346 (0.4) | 0.45 (0.00, 47530.61) | 0.539 |  |
|  |  |  | Placebo/no intervention: 10/1222 (0.8) |  |  |  |
| Trustworthy non-IPD | 2 | 2,631 | Oxytocin: 114/1323 (8.6) | 0.59 (0.18, 1.93) | 0.111 |  |
|  |  |  | Placebo/no intervention: 181/1308 (13.8) |  |  |  |
| Trustworthy Overall | 4 | 5,199 | Oxytocin: 119/ 2669 (4.5) | 0.59 (0.41, 0.84) | 0.017 |  |
|  |  |  | Placebo/no intervention: 191/2530 (7.5) |  |  |  |
| **Oxytocin < 10IU** | | | | | |  |
| Trustworthy IPD | 2 | 1,736 | Oxytocin: 72/877 (8.2) | 0.51 (0.12, 2.15) | 0.106 |  |
|  |  |  | Placebo/no intervention: 116/859 (13.5) |  |  |  |
| Trustworthy non-IPD | 3 | 689 | Oxytocin: 21/302 (7.0) | 0.91 (0.43, 1.91) | 0.638 |  |
|  |  |  | Placebo/no intervention: 31/387 (8.0) |  |  |  |
| Trustworthy Overall | 5 | 2,425 | Oxytocin: 83/1179 (7.0) | 0.65 (0.42, 1.03) | 0.059 | 0.578 |
|  |  |  | Placebo/no intervention: 140/1246 (11.2) |  |  |  |

**Table S9:** Post-hoc sensitivity analysis: oxytocin 10IU vs placebo/no intervention for the prevention of PPH ≥ 1000 mL; oxytocin <10IU vs placebo/no intervention for the prevention of PPH ≥ 1000 mL.

PPH: Postpartum haemorrhage; RCTs: Randomised controlled trials; Pts: Participants; OR: Odds ratio; IPD: Individual participant data; IU: International units.

References

1. Abdel-Aleem H, Singata M, Abdel-Aleem M, Mshweshwe N, Williams X, Hofmeyr GJ. Uterine massage to reduce postpartum hemorrhage after vaginal delivery. International Journal of Gynecology & Obstetrics. 2010;111:32-6.

2. Rosseland LA, Hauge TH, Grindheim G, Stubhaug A, Langesæter E. Changes in blood pressure and cardiac output during cesarean delivery: the effects of oxytocin and carbetocin compared with placebo. Anesthesiology. 2013;119:541-51.

3. Stanton CK, Newton S, Mullany LC, Cofie P, Tawiah Agyemang C, Adiibokah E. Effect on postpartum hemorrhage of prophylactic oxytocin (10 IU) by injection by community health officers in Ghana: a community-based, cluster-randomized trial. PLoS Medicine. 2013;10:e1001524.

4. Jans SMPJ, Herschderfer KC, van Diem MT, Aitink M, Rijnders M, van der Pal-de Bruin K, et al. The LENTE Study: The Effectiveness of Prophylactic Intramuscular Oxytocin in the Third Stage of Labor Among Low-Risk Women in Primary Care Midwifery Practice: A Randomized Controlled Trial. Int J Childbirth. 2016(3):173-82.
